# Intra-Articular Laser Therapy May Be a Feasible Option in Treating Knee Osteoarthritis in Elderly Patients

**DOI:** 10.1155/2022/3683514

**Published:** 2022-11-22

**Authors:** Jean-Lon Chen, Chih-Chin Hsu, Wesley C. C. Chen, Yu-Ning Peng, Carl P. C. Chen, Areerat Suputtitada

**Affiliations:** ^1^Department of Physical Medicine and Rehabilitation, Chang Gung Memorial Hospital at Linkou and College of Medicine, Chang Gung University, Guishan District, Taoyuan City, Taiwan; ^2^Department of Physical Medicine and Rehabilitation, Chang Gung Memorial Hospital at Keelung and College of Medicine, Chang Gung University, Guishan District, Taoyuan City, Taiwan; ^3^Department of Physical Medicine and Rehabilitation, Purple Sun Clinic, Taipei, Taiwan; ^4^Department of Rehabilitation Medicine, Faculty of Medicine, Chulalongkorn University and King Chulalongkorn Memorial Hospital, Bangkok, Thailand

## Abstract

Knee osteoarthritis (OA) is a common problem in elderly patients. They are often troubled with altered knee function, such as pain and weakness. However, not all these patients are able to receive autologous platelet-rich plasma (PRP) injections as they may be taking antiplatelet or anticoagulant medications. Their physical condition may not allow them to receive total knee replacement surgery as well. Long-term oral intake of nonsteroidal anti-inflammatory drugs may be detrimental to the gastrointestinal tract. As a result, it is crucial to discover new treatment options that can alleviate painful knee symptoms in elderly knee OA patients. In this study, 19 elderly patients diagnosed with moderate degree of knee OA as well as suprapatellar bursitis were recruited. They received low-level laser therapy (LLLT) to their affected knees. Under ultrasound guidance, flexible fiber optic wire was inserted intra-articularly into the knee joint. Red laser followed by infrared irradiation was performed once every 2 weeks for a total of 3 times. The Lequesne index for knee OA and the volume of suprapatellar synovial fluid (SF) were measured. SF proteomic analyses were also performed up to a period of 6 months. The results revealed that after 3 LLLT, the Lequesne index significantly decreased, signifying improvement in the knee joint functional status. The volume of suprapatellar SF and SF proteins associated with inflammation also decreased significantly in the SF. These findings lasted up to a period of at least 3 months. Therefore, LLLT may be considered as a feasible option in treating elderly patients with knee OA who are not suitable for surgical interventions or intra-articular PRP injections.

## 1. Introduction

Knee osteoarthritis (OA) is the common cause of knee pain in the elderly population [1, 2]. These patients can be troubled with swelling, pain, and weakness of the knee joint [3]. Cartilage degeneration, osteophyte formation, and knee joint bursitis are the frequent causes [4]. There are many bursae surrounding the knee; suprapatellar bursitis is the likely cause of pain and swelling [5]. This can be diagnosed using musculoskeletal ultrasound or magnetic resonance imaging (MRI) [6, 7]. Knee swelling with pain is positively correlated with the volume of synovial fluid (SF) in the suprapatellar bursa. Pain is often observed when ultrasound image measures greater than 2 millimeters (mm) of bursa distension [5].

Treatment of knee OA can be divided into surgical and nonsurgical options. Surgical intervention such as total knee replacement may not be possible for some patients. For patients who are taking antiplatelet and anticoagulation medications, they may not be able to receive autologous platelet-rich plasma (PRP) injections [6]. Other treatment options include the application of physical modalities, the wearing of insoles, and the injection of other injectants such as micronized dehydrated human amnion/chorion membrane (mdHACM) [8, 9]. Researchers are continuously searching for nonsurgical disease-modifying options for the treatments of knee OA [10]. Due to upper gastrointestinal tract side effects, regular usage of nonsteroidal anti-inflammatory drugs (NSAIDs) in treating knee OA is not recommended [11].

Low-intensity radiation (low-level laser therapy (LLLT)) with specific wavelengths has been known to trigger cellular proliferation and differentiation. The energy density required is very small in LLLT, usually at the level of 2-4 J/cm^2^ and with a transmission power of less than 0.5 watt. The laser light can be absorbed by the chromophores and photoreceptors identified in the respiratory chain of the mitochondria [12]. Laser irradiation in the red or near-infrared spectrum causes mitochondria stimulation. Photoreceptor molecules of the mitochondria absorb laser irradiation as a photostimulant, which then triggers a series of photochemical reactions, causing changes in cellular metabolism such as protein signaling. Through a series of stimulation in the respiratory chain of the mitochondria, adenosine triphosphate (ATP) can be formed. In animal studies, LLLT has been shown to be able to stimulate tissue metabolism and modulate inflammatory pathways in conjunction with PRP [13]. The number of transforming growth factor beta (TGF-*β*) can be increased by LLLT irradiation. TGF-*β* is crucial for cartilage integrity and can act as a powerful tool in preventing or repairing cartilage damage [14].

Studies on the treatment of knee OA using LLLT in humans have reported mixed reports [15]. Some have reported treatment success while some find it ineffective. Ineffective treatment may be caused by the fact that laser irradiation travels a short distance through soft tissues. As a result, treatment effectiveness may be reduced in larger joints such as the knee. Prodromos et al. have suggested that inserting the laser probe from the skin surface into the knee joint can offer better treatment effect to the cartilage and surrounding tissues [15]. The hypothesis of this study is that LLLT can improve the functional status of OA by inserting flexible fiber optic wire intra-articularly into the knee joint. The rheology of SF can be improved, as well as the decrease of protein concentrations associated with the formation of knee OA. As a result, LLLT may be considered as alternative option for elderly knee OA patients who cannot receive surgical interventions or intra-articular (IA) PRP injection treatments.

## 2. Methods

This was a “case-series” study and was conducted in a tertiary hospital. Nineteen elderly patients diagnosed with grade 3 moderate degree of knee OA were recruited. The Kellgren and Lawrence system was used. Their average age was equal or greater than 65 years of age. The Institutional Reviewer Board (IRB) of Chang Gung Medical Foundation approved this study. Patients with history of photoallergy were excluded from this study as they may not be suitable for LLLT. The human research in this study was conducted in accordance with the Declaration of Helsinki.

The inclusion criteria were as follows:

1. Patients unable or did not want to receive surgical treatment such as total knee replacement
2. Had physical conditions that prevented them from receiving anesthesia and surgical treatments
3. Taking antiplatelet or anticoagulant medications that prevented them from receiving treatments such as PRP injections

### 2.1. Treatment Protocol

**Figure 1:**
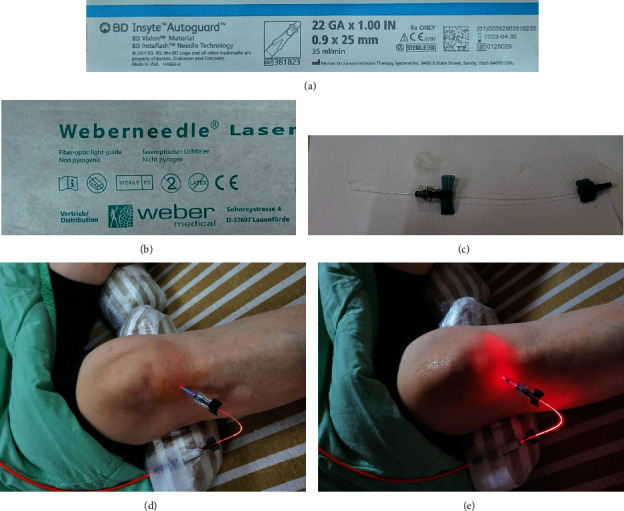
(a) BD Insyte Autoguard 22 GA × 1.00  IN intravenous (IV) hollow catheter was used. (b, c) The Weber nonpyogenic fiber optic light guide. (d) The insertion of the fiber optic light guide was done into the knee joint laterally underneath the patellar tendon. (e) Red laser light diode with a wavelength of 658 nm turned on.

The flexible fiber optic wire was inserted through a hollow needle into the knee joint (BD Insyte Autoguard 22 GA × 1.00  IN intravenous (IV) catheter). This was inserted lateral to the patellar tendon under musculoskeletal ultrasound guidance (Figure 1). The osteoarthritic knee joint received 20 minutes (min) of red (658 nanometer (nm)) laser irradiation followed by 10 min of infrared (810 nm) laser irradiation for a total of 30 min. The intensity of red laser was set at 50 milliwatts (mW), and the intensity of infrared laser was set at 100 mW. LLLT was given once every 2 weeks for a total of 3 times. Before each treatment and at the posttreatment follow-up periods, the Lequesne indices were recorded and knee SF aspirated. The aspirated SF was measured for volume and sent for proteomic analyses. The posttreatment follow-up periods were at 1, 3, and 6 months after 3 LLLT treatment courses.

**Figure 2:**
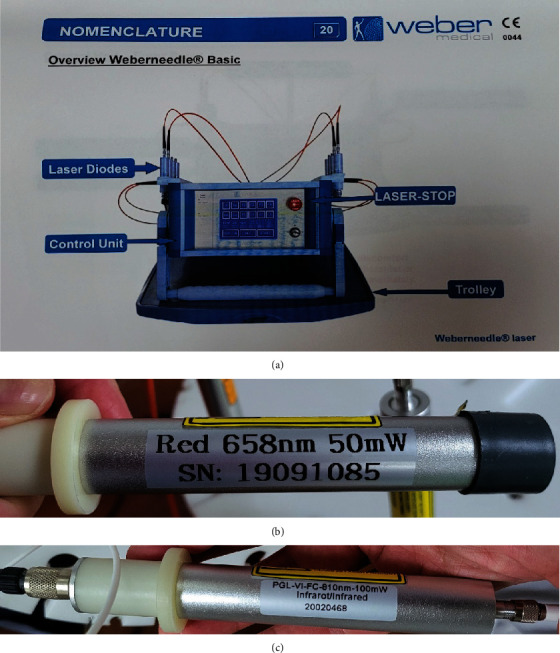
(a) Weberneedle® basic laser device was used in this study. Red and infrared laser diodes were used. (b) Red laser light diode with a wavelength of 658 nm and with a maximum intensity of 50 mW. (c) Infrared laser light diode with a wavelength of 810 nm and with a maximum intensity of 100 mW.

The low-power laser machine used in this study was the Weberneedle® basic laser device (Weber Medical GmbH, Germany) (Figure 2). The 2 laser diodes were the red laser light with a wavelength of 658 nm with a maximum intensity of 50 mW and the infrared laser light with a wavelength of 810 nm with a maximum intensity of 100 mW.

### 2.2. The Lequesne Index

The Lequesne index for degenerative OA knee joint was used in this study to evaluate the extent of knee functional status before, during, and after LLLT [16]. The same experimenter performed all the Lequesne index evaluations to avoid intertester variability. Knee OA is likely when the Lequesne index value is greater than 7 [17].

### 2.3. The Aspiration of Knee Joint Synovial Fluid

**Figure 3:**
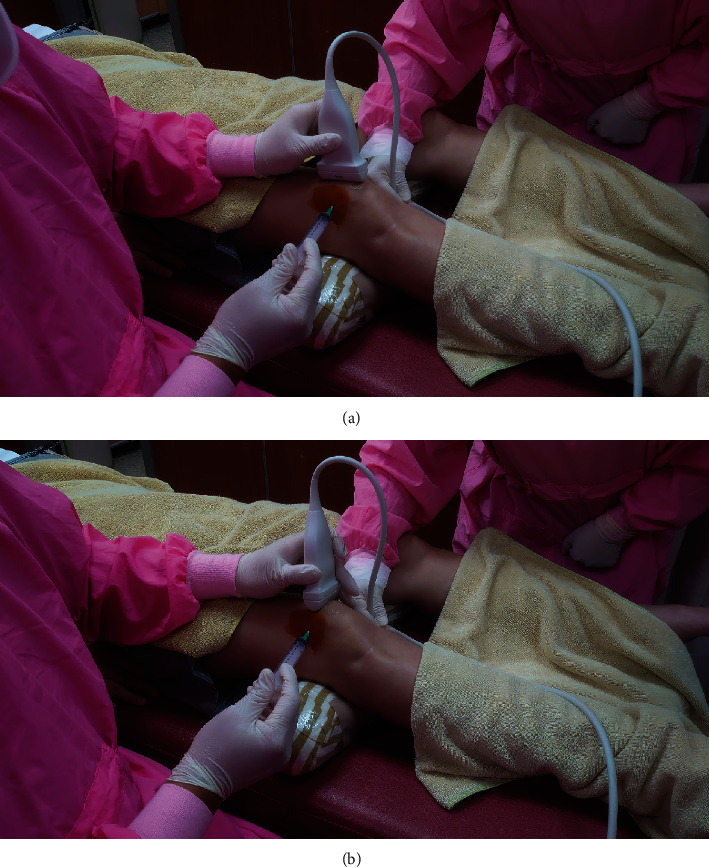
With the knee flexed slightly and a pillow placed underneath the joint, the standard lateral approach was performed for the aspiration procedure. (a) Needle inserted under the transverse view of the transducer. (b) Needle inserted under the longitudinal view of the transducer.

Musculoskeletal soft tissue ultrasound was used in performing accurate aspiration and fiber optic insertion procedures. The T3300 tablet ultrasound imaging system was used (BenQ Medical Technology Corporation). The linear transducer bandwidth was 4–15 MHz. Accurate ultrasound-guided needle placement into the bursa is required to prevent poking into the soft tissues and vessels to avoid SF blood contamination. The aspiration procedure was done under the standard lateral approach. Under this approach, the knee was slightly flexed with a pillow placed underneath the joint (Figure 3). In order to avoid experimental bias, the same physician performed the aspiration procedure and ultrasound image interpretation. The volume of the aspirated SF was then recorded.

### 2.4. Proteomic Technique of 2-Dimensional Electrophoresis

#### 2.4.1. Sample Preparation

After aspiration, SF samples were placed immediately on ice and processed within the hour. SF samples contaminated with visible blood were discarded. Two-dimensional electrophoresis (2-DE) was used in this study as the tool for proteomic analysis. Hundreds of protein spots can be identified in one 2-DE gel. Under the 1^st^ dimension, proteins are separated according to the charges or isoelectric points (pI). The process of protein separation according to their pI is called isoelectric focusing (IEF) [18]. The second dimension is the separation of the focused proteins based on their relative molecular weights (Mr). The Bio-Rad PDQuest 2-D analysis software was used for protein spot analysis on 2-DE gel images. After image analysis on the protein spots, the ones that revealed significant intensity differences were excised from the SYPRO Ruby stained gels in order to conduct further mass spectrometry (MS) to identify their correct protein names.

#### 2.4.2. Statistical Analysis

In order to assure that the data in this study was normally distributed, the Kolmogorov-Smirnov was used. The Wilcoxon signed-rank test was used to compare the age differences between patients. Repeated measures ANOVA with the Bonferroni correction was used for the comparison of the Lequesne index values, SF volume, and SF protein concentrations before, during, and after LLLT. These parameters were measured during 1^st^ LLLT, 2^nd^ LLLT, 3^rd^ LLLT, and posttreatment follow-up periods of 1, 3, and 6 months. SPSS software was applied for the statistical comparisons in this study. *P* values less than 0.05 were considered significant statistically.

## 3. Results

In the recruited 19 patients (14 female and 5 male patients), the mean age was 68 ± 2.4 years and without significant statistical differences. The aspirated SF volume was measured to have an average of 16.44 ± 3.96 mL prior to the 1^st^ LLLT. The average aspirated SF volumes were then measured to be 15.51 ± 3.61 mL at the time of 2^nd^ LLLT and 5.78 ± 2.43 mL at the time of 3^rd^ LLLT. At 1 month after the completion of LLLT, the measured average aspirated SF volume was 6.38 ± 3.04 mL. At 3 months after the completion of LLLT, the measured average aspirated SF volume was 7.58 ± 3.29 mL. At 6 months after the completion of LLLT, the measured average aspirated SF volume was 9.22 ± 3.46 mL (Table 1).

In the Lequesne index for knee OA assessment, the average value was measured to be 13.31 ± 4.11 prior to the 1^st^ LLLT. Index values were then recorded to have average values of 12.44 ± 2.79 at the time of 2^nd^ LLLT and 4.47 ± 2.33 at the time of the 3^rd^ LLLT. At 1 month after the completion of LLLT, the index value was measured to have an average of 4.05 ± 2.14. At 3 months after the completion of LLLT, the index value was measured to have an average of 4.74 ± 2.22. At 6 months after the completion of LLLT, the index value was measured to have an average of 6.15 ± 2.78 (Table 1).

**Figure 4:**
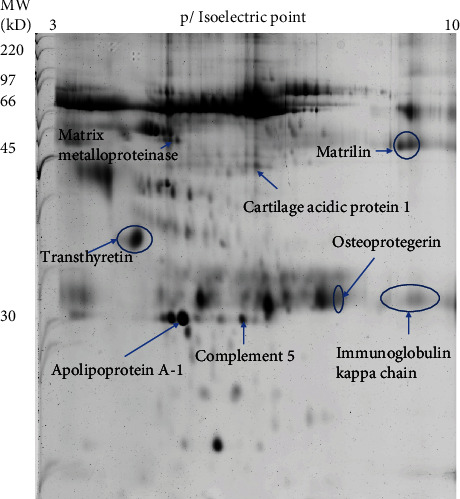
Synovial fluid two-dimensional electrophoresis gel image of proteins spots. Proteins were identified by mass spectrometry. MW signifies molecular weight and is expressed in KD (kilodaltons).

Out of the hundreds of protein spots analyzed on the SF 2-DE gel images, 8 proteins that revealed the highest intensity changes (ex/more than 2-folds) were chosen for further literature search on its pathophysiological functions. Four proteins that showed greater than 2-fold increase in protein concentrations (intensities) were transthyretin (TTR), osteoprotegerin (OPG), complement 5 (C5), and matrilin (MATN). Cartilage acidic protein 1 (CRTAC1), matrix metalloproteinase (MMP), apolipoprotein A-1 (APOA1), and immunoglobulin kappa chain (IGKC) were the 4 proteins that revealed significant 2-fold decrease in protein intensities (SF protein names are shown in Table 2, and protein spots on 2-DE gels are shown in Figure 4).

## 4. Discussion

This study is aimed at finding out whether intra-articular (IA) placement of flexible fiber optic wire low-level laser therapy (LLLT) is effective in treating patients with knee osteoarthritis (OA). The results in this study showed that after at least 2 LLLT treatments, SF protein concentrations associated with the attenuation of cartilage damage increased, knee joint functional status improved, and SF volumes and inflammatory proteins decreased significantly. The reason that LLLT was performed at 2 weeks apart was because 2 to 3 IA knee platelet-rich plasma (PRP) injection courses are recommended to treat patients with knee OA. Each injection is performed at 2 to 3 weeks apart [19]. Therefore, the results obtained in this study can be compared with other PRP studies to see if LLLT can be equally effective.

The WOMAC osteoarthritis index and Lequesne index can be used for the evaluation of knee OA functional status [20]. Both indices are reliable instruments for the evaluation of OA. In this study, the Lequesne index was chosen as the evaluation tool instead of WOMAC because it has less items to evaluate. Therefore, less time is required during the evaluation process. After 2 LLLT treatments and at the time of 3^rd^ LLLT, the Lequesne index value decreased significantly to 4.47 ± 2.33 as compared with no LLLT (13.31 ± 4.11) or received only 1 LLLT (12.44 ± 2.79). An index value of higher than 7 is suggestive of OA [16, 21]. Therefore, this significant decrease in the Lequesne index suggested that 2 LLLT treatment courses, each at 2 weeks apart, can improve knee functional status in OA patients for about 3 months.

The severity of suprapatellar bursitis is related to the degree of cartilage loss. Higher suprapatellar bursa SF volumes are associated with more severe cartilage loss in the knee joints [22]. Therefore, without the presence of bursitis, aspiration of adequate knee SF for proteomic study may be difficult. After 2 LLLT treatments, the volume of SF decreased significantly, implying that the process of cartilage loss or degradation may be attenuated.

Through 2-DE gel imaging analytical software, changes in the protein concentrations can be calculated. The higher the intensity of a protein spot on the gel image, the higher the concentration [17, 23]. Instead of listing all the changes in protein concentrations, this study chose 8 SF proteins that depicted the highest changes in concentrations after LLLT. Thorough literature search on the pathophysiological functions of these proteins was then conducted. Four proteins with at least 2-fold increase in concentrations were MATN, OPG, TTR, and C5. MATN is an extracellular and noncollagenous matrix protein. These proteins have been shown to play crucial roles in regulating the microenvironment of the cartilage. Chondrocyte hypertrophy can be observed in MATN knockout mice, indicating degeneration of the cartilage. Therefore, increased MATN concentration after LLLT suggests possible inhibition in chondrocyte hypertrophy, further attenuating cartilage degeneration [24]. The complement system is regulated by inflammatory and catabolic mediators in the development of OA [25]. The C5 protein plays a key position in degenerative joint disorders. Activation of the complement system can contribute to tissue repair by chemoattraction of stem and progenitor cells [26]. In this study, the concentration of C5 was significantly increased after LLLT, which may imply that cartilage angiogenesis and stem cell recruitment are initiated.

OPG protein is produced by osteoblasts. Its main function is the inhibition of osteoclastogenesis by binding to receptor activator of nuclear factor kappa-*Β* ligand (RANKL). Evidence has shown that OPG/RANKL ratio is altered in knee OA and is associated with subchondral bone alterations [27]. Significant increase in OPG concentration was noted after LLLT, which may imply that bone resorption can be inhibited, and less alteration of OPG/RANKL ratio can be achieved. The majority of TTR, an amyloidogenic protein, is produced in the liver and in the choroid plexus. TTR can be found in the aging knee joint cartilage surface [28]. The development of knee OA includes the risk factor of amyloid deposition. TTR is capable of chelating amyloid and further preventing its accumulation within the knee joint [17]. Therefore, significant SF increase in TTR concentration by using LLLT may imply that the development of knee OA can be attenuated.

CRTAC1 concentration was significantly decreased after LLLT. Through proteomic analysis, elevated CRTAC1 was observed in the SF of patients with OA. It was observed that genetic deletion of CRTAC1 in mice can result in the inhibition osteophyte formation and cartilage degradation [29]. Therefore, decreased CRTAC1 may suggest that the degree of cartilage degradation can be reduced in OA patients after LLLT. Knee joint inflammation may lead to OA and is associated with the dysregulation of lipid profile. Plasma-derived APOA1 has the physiological role of lipid transport and delivery. APOA1 has proinflammatory properties and is associated with increased matrix metalloproteinase (MMP) expressions [30]. Increased expression of MMPs can aggravate joint cavity inflammation, resulting in decreased expression of chondrocyte proteins. These will cause irreversible dysfunction in OA, resulting in painful knee and loss of joint movement [31]. IGKC is secreted by B lymphocytes and is a type of light chain associated with the production of antibody. In inflammatory joint, increased level of IGKC can be seen [32]. Therefore, significant drops of APOA1, MMP, and IGKC concentrations may imply that inflammation may be decreased in the knee joint with LLLT.

In summary, 20 min of red (658 nm) laser irradiation followed by 10 min of infrared (810 nm) laser irradiation for a total of 30 min into the knee joint can provide positive treatment effectiveness in OA patients. The rationale behind this therapy may be that growth factors such as TGF-*β* are induced after irradiation. TGF-*β* assists in maintaining cartilage integrity and can repair the damaged cartilages [14]. The activation of phagocytic activity of macrophages can also be observed after irradiation [33]. In this study, protein associated with chelation (ex/TTR) was significantly increased, suggesting that the rheology of SF can be improved after LLLT. After 3 LLLT, SF protein concentrations associated with cartilage degeneration and inflammation were significantly decreased. A decrease in the Lequesne index to a value less than 7 suggested that 3 LLLT treatments can significantly improve knee function in OA patients. Based on the resulted obtained in this study, at least 2 LLLT are needed in order to offer beneficial outcome for the treatment of knee OA. The effect can last for at least 3 months. Therefore, LLLT can be considered as one of the feasible treatment options for elderly knee OA patients when surgery and IA PRP injections are not suitable for them.

## 5. Conclusion

Low-level laser therapy (LLLT) using 20 min of red (658 nm) laser irradiation followed by 10 min of infrared (810 nm) laser irradiation for a total of 30 min into the knee joint has shown to be beneficial for the treatment of knee OA (moderate degree) in elderly patients. SF rheology and knee joint functional status are improved after at least 2 LLLT treatments. SF proteins associated with cartilage inflammation and degeneration can be significantly decreased after LLLT treatments, indicating that the process of OA may be attenuated. Treatment effectiveness can last up to a period of at least 3 months. Therefore, LLLT can be a feasible option in treating elderly patients with moderate degree of knee OA when surgery and intra-articular PRP injections are not preferred by these patients. LLLT may be repeated again after 3 months.

## Figures and Tables

**Table 1:** Changes in the parameters of the Lequesne index and aspirated synovial fluid volumes before, during, and after LLLT.

	Before LLLT (time of 1^st^ LLLT)	2 weeks after 1^st^ LLLT (time of 2^nd^ LLLT)	2 weeks after 2^st^ LLLT (time of 3^rd^ LLLT)	1-month follow-up	3-month follow-up	6-month follow-up
Average aspirated SF volume in mL	16.44 ± 3.96	15.51 ± 3.61	5.78 ± 2.43^∗^	6.38 ± 3.04^∗^	7.58 ± 3.29^∗^	9.22 ± 3.46
Lequesne functional index	13.31 ± 4.11	12.44 ± 2.79	4.47 ± 2.33^∗^	4.05 ± 2.14^∗^	4.74 ± 2.22^∗^	6.15 ± 2.78

LLLT: low-level laser therapy; mL: milliliter. ^∗^Statistical analysis of 3^rd^ LLLT (at this time, patients have completed 2 LLLT treatments), 1-month follow-up, and 3-month follow-up periods as compared with the time before LLLT or received only 1 LLLT and at 6 months follow-up (*p* < 0.05). Values are expressed as mean ± standard deviation (SD).

**Table 2:** Representative synovial fluid proteins with the highest concentration changes after LLLT.

Spot name^a^	Access no^b^	pI^c^	Mr (kDa)^d^
Matrilin (MATN)	P21941	8.31	54.00
Transthyretin (TTR)	P02787	6.81	79.29
Complement 5 (C5)	P06684	8.90	112.75
Osteoprotegerin (OPG)	Q80VU4	8.70	34.00
Apolipoprotein A-1 (APOA1)	P02647	5.56	30.00
Immunoglobulin kappa chain (IGKC)	P01834	9.10	11.61
Cartilage acidic protein 1 (CRTAC1)	Q9NQ79	5.20	68.00
Matrix metalloproteinase (MMP)	P14780	5.70	92.00

^a, b^Protein names and accession numbers as searched the databases of TrEMBL and Swiss-Prot. ^c^Isoelectric point (pI) of the protein. ^d^The protein molecular weight as expressed in kilodaltons.

## Data Availability

All the data are expressed in detail in Tables 1 and 2. Further data can be available upon request.

## References

[1] Kon E., Mandelbaum B., Buda R. (2011). Platelet-rich plasma intra-articular injection versus hyaluronic acid viscosupplementation as treatments for cartilage pathology: from early degeneration to osteoarthritis. *Arthroscopy: The Journal of Arthroscopic & Related Surgery*.

[2] Yagi S., Sata M. (2019). Rupture of pes anserine bursa in a patient with pes anserine pain syndrome due to osteoarthritis. *The Journal of Medical Investigation*.

[3] Sari Z., Aydoğdu O., Demirbüken İ., Yurdalan S. U., Polat M. G. (2019). A better way to decrease knee swelling in patients with knee osteoarthritis: a single-blind randomised controlled trial. *Pain Research and Managemen*.

[4] Peterfy C. G., Guermazi A., Zaim S. (2004). Whole-organ magnetic resonance imaging score (WORMS) of the knee in osteoarthritis. *Osteoarthritis Cartilage*.

[5] de Miguel Mendieta E., Ibáñez T. C., Jaeger J. U., Hernán G. B., Mola E. M. (2006). Clinical and ultrasonographic findings related to knee pain in osteoarthritis. *Osteoarthritis Cartilage*.

[6] Beaman F. D., Peterson J. J. (2007). MR imaging of cysts, ganglia, and bursae about the knee. *Magn Reson Imaging Clin N Am*.

[7] Chen C. P. C., Hsu C. C., Huang S. C., Lin M. Y., Chen J. L., Lin S. Y. (2020). The application of thermal oscillation method to augment the effectiveness of autologous platelet rich plasma in treating elderly patients with knee osteoarthritis. *Experimental Gerontology*.

[8] Alden K. J., Harris S., Hubbs B., Kot K., Istwan N. B., Mason D. (2021). Micronized dehydrated human amnion chorion membrane injection in the treatment of knee osteoarthritis-a large retrospective case series. *The Journal of Knee Surgery*.

[9] Page C. J., Hinman R. S., Bennell K. L. (2011). Physiotherapy management of knee osteoarthritis. *International Journal of Rheumatic Diseases*.

[10] Huang Z., Chen J., Ma J., Shen B., Pei F., Kraus V. B. (2015). Effectiveness of low-level laser therapy in patients with knee osteoarthritis: a systematic review and meta-analysis. *Osteoarthritis Cartilage*.

[11] Bijlsma J. W., Berenbaum F., Lafeber F. P. (2011). Osteoarthritis: an update with relevance for clinical practice. *The Lancet*.

[12] Alves A. C., Vieira R., Leal-Junior E. (2013). Effect of low-level laser therapy on the expression of inflammatory mediators and on neutrophils and macrophages in acute joint inflammation. *Arthritis Research & Therapy*.

[13] Karu T. I., Pyatibrat L. V., Afanasyeva N. I. (2005). Cellular effects of low power laser therapy can be mediated by nitric oxide. *Lasers in Surgery and Medicine*.

[14] Blaney Davidson E. N., van der Kraan P. M., van den Berg W. B. (2007). TGF-*β* and osteoarthritis. *Osteoarthritis Cartilage*.

[15] Prodromos C. C., Finkle S., Dawes A., Dizon A. (2019). Intra-articular laser treatment plus platelet rich plasma (PRP) significantly reduces pain in many patients who had failed prior PRP treatment. *Medicines*.

[16] Lequesne M. G. (1997). The algofunctional indices for hip and knee osteoarthritis. *J Rheumatol*.

[17] Chen C. P. C., Cheng C. H., Hsu C. C., Lin H. C., Tsai Y. R., Chen J. L. (2017). The influence of platelet rich plasma on synovial fluid volumes, protein concentrations, and severity of pain in patients with knee osteoarthritis. *Experimental Gerontology*.

[18] Rabilloud T., Adessi C., Giraudel A., Lunardi J. (1997). Improvement of the solubilization of proteins in two-dimensional electrophoresis with immobilized pH gradients. *Electrophoresis*.

[19] Kavadar G., Demircioglu D. T., Celik M. Y., Emre T. Y. (2015). Effectiveness of platelet-rich plasma in the treatment of moderate knee osteoarthritis: a randomized prospective study. *Journal of Physical Therapy Science*.

[20] Nadrian H., Moghimi N., Nadrian E. (2012). Validity and reliability of the Persian versions of WOMAC osteoarthritis index and Lequesne algofunctional index. *Clinical Rheumatology*.

[21] Lecorney J., Verhoeven F., Chouk M., Guillot X., Prati C., Wendling D. (2018). Correlation between catastrophizing and Lequesne index in case of osteoarthritis of the knee: a prospective study. *Joint Bone Spine*.

[22] Heidari B., Hajian-Tilaki K., Babaei M. (2016). Determinants of pain in patients with symptomatic knee osteoarthritis. *Caspian J Intern Med*.

[23] Chen C. P., Hsu C. C., Yeh W. L. (2011). Optimizing human synovial fluid preparation for two-dimensional gel electrophoresis. *Proteome Science*.

[24] Yang X., Trehan S. K., Guan Y. (2014). Matrilin-3 Inhibits Chondrocyte Hypertrophy as a Bone Morphogenetic Protein-2 Antagonist. *Journal of Biological Chemistry*.

[25] Silawal S., Triebel J., Bertsch T., Schulze-Tanzil G. (2018). Osteoarthritis and the complement cascade. *Clinical Medicine Insights: Arthritis and Musculoskeletal Disorders*.

[26] Schraufstatter I. U., Khaldoyanidi S. K., DiScipio R. G. (2015). Complement activation in the context of stem cells and tissue repair. *World Journal of Stem Cells*.

[27] Tat S. K., Pelletier J. P., Velasco C. R., Padrines M., Martel-Pelletier J. (2009). New perspective in osteoarthritis: the OPG and RANKL system as a potential therapeutic target?. *The Keio Journal of Medicine*.

[28] Akasaki Y., Reixach N., Matsuzaki T. (2015). Transthyretin deposition in articular cartilage: a novel mechanism in the pathogenesis of osteoarthritis. *Arthritis Rheumatol*.

[29] Ge X., Ritter S. Y., Tsang K., Shi R., Takei K., Aliprantis A. O. (2016). Sex-specific protection of osteoarthritis by deleting cartilage acid protein 1. *PLoS One*.

[30] Oliviero F., Sfriso P., Baldo G. (2009). Apolipoprotein A-I and cholesterol in synovial fluid of patients with rheumatoid arthritis, psoriatic arthritis and osteoarthritis. *Clin Exp Rheumatol*.

[31] Lu S., Xiao X., Cheng M. (2015). Matrine inhibits IL-1*β*-induced expression of matrix metalloproteinases by suppressing the activation of MAPK and NF-*κ*B in human chondrocytes in vitro. *Int J Clin Exp Pathol*.

[32] Chang X., Cui Y., Zong M. (2009). Identification of proteins with increased expression in rheumatoid arthritis synovial tissues. *The Journal of Rheumatology*.

[33] Gulsoy M., Ozer G. H., Bozkulak O. (2006). The biological effects of 632.8-nm low energy He-Ne laser on peripheral blood mononuclear cells in vitro. *Journal of Photochemistry and Photobiology B: Biology*.

